# Genome assembly of the Korean intertidal mud-creeper *Batillaria attramentaria*

**DOI:** 10.1038/s41597-023-02403-9

**Published:** 2023-07-28

**Authors:** Ajit Kumar Patra, Phuong-Thao Ho, Siyeong Jun, Seung Jae Lee, Yuseob Kim, Yong-Jin Won

**Affiliations:** 1 Department of Life Science, Division of EcoScience, Ewha Womans University, Seoul, South Korea; 2 Laboratory of Ecology and Environmental Management, Science and Technology Advanced Institute, Van Lang University, Ho Chi Minh City, Vietnam; 3 Department of International Program, US Vietnam Talent International School, Ho Chi Minh city, Viet Nam; 4 Bioinformatics Team, DNA Link, Seoul, South Korea

**Keywords:** Phylogenomics, Invasive species

## Abstract

Batillaridae is a common gastropod family that occurs abundantly in the shallow coastal zone of the intertidal mudflats of the northwest Pacific Ocean, Australasia, and North America. In this family, *Batillaria attramentaria* is known for its biological invasion and colonization in estuarine and intertidal zones. It can endure and adapt the harsh intertidal conditions such as frequent temperature alteration, salinity, and air exposure. Therefore, we sequenced and assembled this Korean batillariid genome to get insight into its intertidal adaptive features. Approximately 53 Gb of DNA sequences were generated, and 863 scaffolds were assembled into a draft genome of 0.715 Gb with 97.1% BUSCO completeness value. A total of 40,596 genes were predicted. We estimated that *B. attramentaria* and *Conus consors* diverged about 230 million years ago (MYA) based on the phylogenetic analysis of closely related gastropod species. This genome study sets the footstep for genomics studies among native and introduced *Batillaria* populations and the Batillaridae family members.

## Background & Summary

Batillariidae, also called batillariids or mud-creepers are widely distributed in the north-western Pacific region of Asia along the complex coastline formed in Japan, Korea, eastern China, and America^1–4^. Within the Batillaridae family, *B. attramentaria* (Sowerby, 1855) is characterized by its habitats being limited to narrow intertidal zones consisting of rocks or sandy mud along coastlines and limited dispersal capacity associated with direct larval development^5,6^. Due to such biological constraints, geographical movement distance is limited, and its population structure is also inferred to be influenced by geographical factors^6–8^. These characteristics hinder them from escaping from their originated habitats. However, in the early 20^th^ century, *B. attramentaria* was introduced into the Bay of the Northeast Coast in the United States and Canada by commercial shipment of oyster (*Crassostrea gigas*) aquaculture from Japan^9,10^. In the new habitat, this invaded species not only flourished but also successfully competed with the native gastropod species such as *Cerithidea californica*^11–14^.

The mitochondrial lineage of *B. attramentaria* is primarily subdivided into two, and their geographical distribution matches the trajectories of two dominant regional seawater currents, Tsushima and Kuroshio, that flow separately north and south of the Japanese archipelago^2^. An analysis of the demographic history of *B. attramentaria* indicates that this species has sharply increased approximately since the last glacial maximum (LGM: 26,000–19,000 years ago), directly influenced by the sea level rise and range expansion of habitat in Asia following climate change^1^.

Benthic organisms living in the estuarine intertidal zone are subjected to the most dynamic environmental circumstances, with frequently altered salinity and temperature in their habitat due to tidal conditions. Thus, estuarine intertidal organisms are continuously exposed daily to the submerged saline seawater and cold temperature during high tide and to the dry, low salinity and high temperature during the low tide. Subsequently, continuous exposure to such highly variable environmental conditions has shaped intertidal communities’ behavioural and physiological adaptation and genetic variation^15,16^. Salt stress exposure study on *B. attramentaria* shows that variation in salinity affects their locomotion activity^17^, which seems to be a typical response observed in several intertidal gastropods^18–20^. Among several studies of molluscs, a survey on intertidal oyster *Crassostrea gigas* highlights the pathways and genes involved in responding to and adapting to typical tidal environmental conditions^17^. In comparison, a study on terrestrial giant African snails shows the expansion of mucus-related gene families to mitigate dry conditions on the land and the doubling of several genes, including haemocyanin (a copper-containing respiratory protein) that helps in transporting oxygen and phosphoenolpyruvate carboxykinase gene families during whole genome duplication^21^. Adaptation to such typical intertidal and terrestrial environmental conditions was achieved by regulating water balance, air-breathing, nitrogen excretion, neural–immune system interactions, and specific behaviours.

In this context, the genome sequence of *B. attramentaria* will be beneficial for a deepened understanding of its evolution and invasiveness. It could be a suitable model for studying the combined influence of climate change and palaeoceanographic change on marine gastropods and other coastal taxa in the Northeast Asian region. As well as this study will enrich our knowledge of the genetic features involved in the adaptation to typical intertidal environmental factors.

Here, we present a first draft of reference genome assembly for *B. attramentaria* constructed using long reads generated by the Pacific Biosciences (PacBio) DNA sequencing platform Sequel and short paired-end reads generated by Illumina. The genome was assembled into 863 scaffolds (N50 = 1.28 Mb), with a total size of 0.715 Gb, with 97.2% assembly completeness analysed by BUSCO. The genome completeness is on par with the mollusc genomes sequenced to date. Structural annotation of the genome yielded 40,596 genes. Of the total genes predicted, 15,755 genes were functionally annotated with InterProScan. Based on phylogenetic analysis of related gastropod species, *B. attramentaria* diverged from *Conus consors* during the Early Mesozoic era, i.e., about 230 MYA. We have detected genes responsible for adapting to intertidal environments^22^ (Supplementary Table 1) such as the Na^+^/H^+^ exchanger family, Na^+^/K^+^ ATPase (for ionic regulation), acyltransferase, proline dehydrogenase (for osmotic regulation), haemocyanin beta-sandwich, animal haem peroxidase, protein-tyrosine phosphatase (for improving terrestrial respiratory function), and galactosyltransferase, Ependymin, TNF(Tumour Necrosis Factor) family, C1q domain (for immune defense), as observed in terrestrial and marine gastropods in previous studies^15,16,21,22^.

## Methods

### Sample collection and purification of DNA

To construct a draft of the reference genome for the Korean batillariids, we collected samples from *B. attramentaria* (Sowerby, 1855) from Hajeon-ri, Cheollabuk-do, South Korea (on November 2018 at 35°32′N, 126°33′E). The samples were kept alive in seawater during the transportation to the laboratory. To obtain high quality and molecular weight of DNA, we dissected fresh tissues from the foot to muscle part of the alive samples and quickly froze them at −80 °C. We did not include the gut part to avoid the snail’s intestinal microbiome contaminant to the snail DNA. Genomic DNA was extracted using the Dneasy® Blood & Tissue kit (Qiagen, Hilden, Germany), and the integrity was checked using an agarose gel.

### Short-read DNA sequencing and genome size estimation

**Fig. 1:**
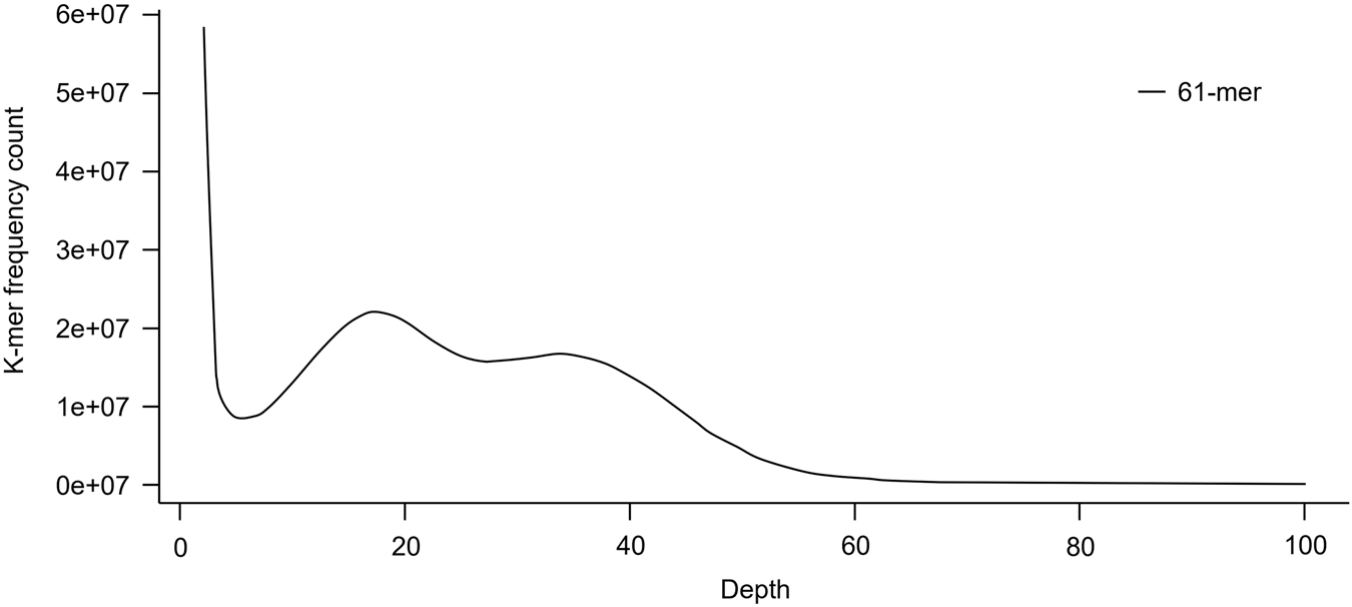
*B. attramentaria* genome size estimation by k-mer distribution.

We constructed a library with an insert size of 350 bp using a Truseq Nano DNA Library kit (Illumina, SD, USA) following random fragmentation and adaptor ligation to DNA sequences. Paired-end (PE) sequencing with 101 bp was carried out using the Hiseq. 4000 sequencing system (Illumina, CA, USA), which generated a total of 731,221,132 PE reads (73.9 Gbp) (Supplementary Table 2). The JELLYFISH tool^23^ was used to estimate the genome size of *B. attramentaria*, which resulted in approximately 0.64 Gbp based on k-mer distribution value (K = 61). The main peak at k-mer depth 34 was used for genome size estimation (Fig. 1).

### PacBio sequencing

The genomic DNA was sheared to generate ~20Kb fragments using the Covaris g-TUBE (Covaris) according to the manufacturer’s instructions. Small fragments were removed by the AMpureXP bead purification system (Beckman Coulter). A total of 5 μg DNA for each sample was used to prepare the library using SMRTbell® Express Template Prep Kit v2.0 (Pacific Biosciences, Menlo Park, CA, USA). Small fragments were removed from the library by BluePippin Size selection system for the large-insert library. Then the sequencing primer v4 was annealed to the SMRTbell template, followed by the binding of DNA polymerase to the complex (Sequel Binding kit 3.0). The excess primer and polymerase were removed from the complex using AMPure purification system before sequencing. Finally, the SMRT library was sequenced using the PacBio Sequel System with the Sequel Sequencing Kit v3.0 chemistry. A total of ~53.3 Gbp of subreads were obtained (Supplementary Table 3).

### Genome assembly and polishing

Initially, cleaned PacBio long-read sequences were assembled using FALCON-Unzip assembler^24^, which generated a contiguous assembly of 844 Mbp (N50 = 1.08 Mbp). The larger size of the assembly than the estimated genome size suggested a high number of duplicate haplotypes^25^. The highly heterozygous genome assembly was curated by Purge Haplotigs^26^ to generate a de-duplicated haploid genome assembly. Further, the assembled genome was polished by Pilon 1.2.3 (with default parameters)^27^ by using aligned Illumina PE reads (57.5 Gb), resulting in a final assembly of 863 contigs with a total length of 715 Mb and an N50 length of 1.28 Mb (Table 1).

**Table 1:** Statistics of genome assembly of *B. attramentaria*.

	FALCON-Unzip	Purge_haplotigs	Final
Number of contigs	1,758	863	863
Total size of contigs	844,056,538	717,569,005	715,684,482
Longest contig size	9,185,056	9,185,056	9,161,156
Number of contigs >1 K nt	1,758	863	863
Number of contigs >10 K nt	1,758	863	863
Number of contigs >100 K nt	1,257	799	799
Number of contigs >1 M nt	243	242	241
Mean contig size	480,123	831,482	829,298
Median contig size	204,571	575,946	570,111
N50 contig length	1,079,716	1,290,776	1,288,332
L50 contig count	212	158	158
GC Contents (%)	45	45	45

**Fig. 2:**
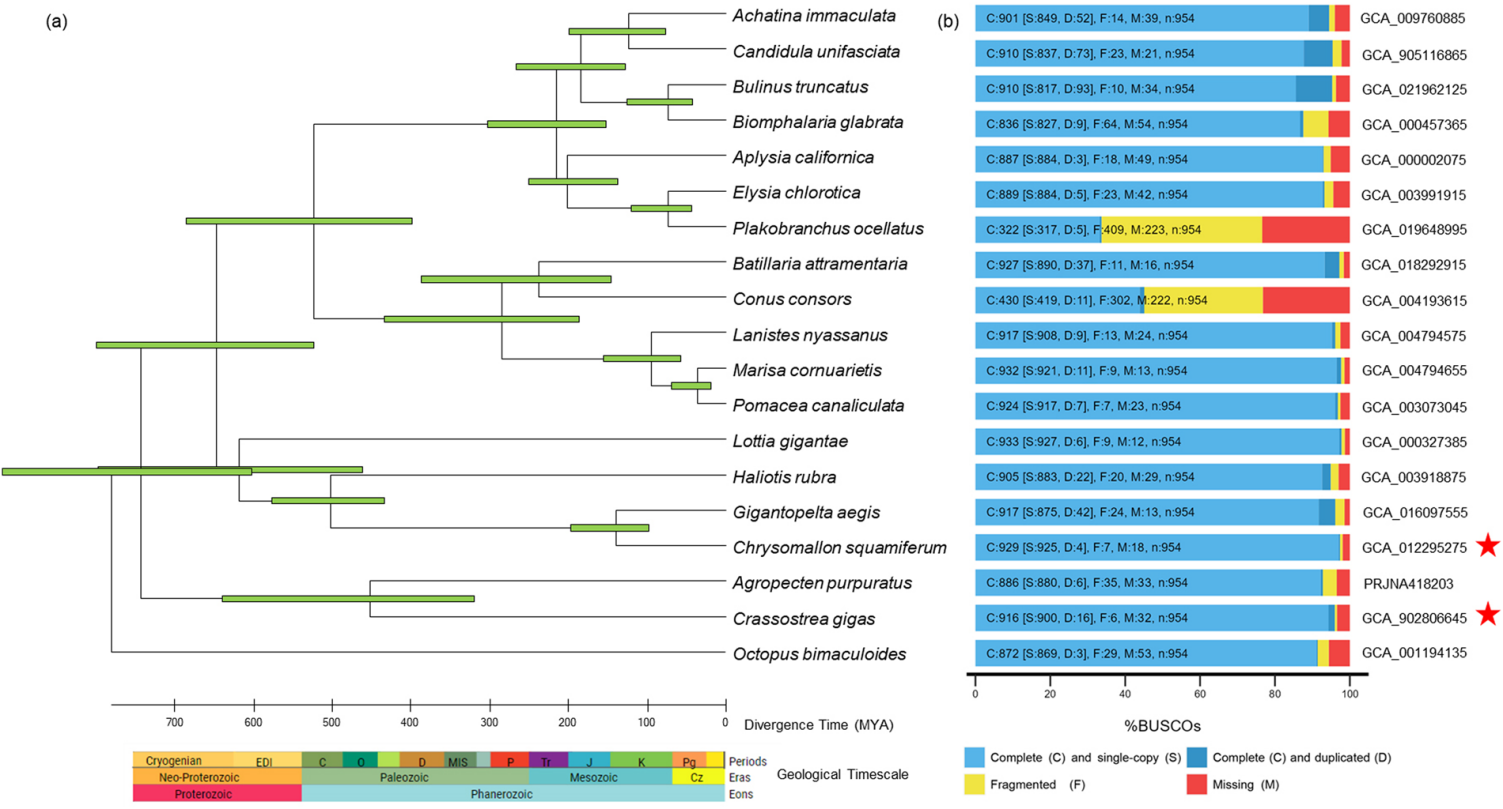
(**a**) Divergence time tree among gastropods. Divergent times were estimated using RelTime methods with an ML phylogenomic tree, and the unit of time was scaled in millions of years. Bars around each node represent 95% confidence intervals. (**b**) Genome assembly completeness comparison estimated by BUSCO (metazoan_odb10). The genome assembly accession number is mentioned next to the plot. Assembly of *A. purpuratus* was analyzed from http://gigadb.org/dataset/100419. Star marks represent the chromosome-level assemblies.

The assembled genome is much smaller than the closest sequenced genome of Conus consors (3.025 Gb)^28^. Due to high heterozygosity levels and repetitiveness, the assembly processes of molluscs are found to be complicated. Such instances were observed in oysters and other invertebrates^29^. The repeat content was estimated to be (314 Mb) 43.87% of the genome assembled (Table 2). Most invertebrate genomes, including molluscs, exhibit high heterozygosity and repetitiveness, complicating the assembly process. Genome completeness estimated by using BUSCO (Benchmarking Universal Single-Copy Orthologs) v3.0.2 detected a total of 927 (97.2%) of the 954 genes in the metazoan gene set^30^ (Table 3). Genome completeness is par with other mollusc genome assembly available till date (Fig. 2b)

**Table 2:** Statistics of repeat elements of *B. attramentaria*.

	Number of elements	Length occupied (bp)	Percentage of sequences (%)
SINEs	231,812	39,739,927	5.55
LINEs	137,704	34,564,778	4.83
LTR elements	48,216	7,903,640	1.1
DNA elements	156,856	27,301,116	3.81
Unclassified	787,378	146,106,066	20.41
Small RNA	73,161	10,762,653	1.5
Satellites	37,148	4,270,032	0.6
Simple repeats	909,136	52,830,592	7.38
Low complexity	68,230	3,915,780	0.55
Total		313,966,700	43.87

**Table 3:** BUSCO assessment of *B. attramentaria* genome assembly (Metazoa).

	Number of BUSCOs	Percentage of BUSCOs
Complete BUSCOs	950	97.1
Complete Single-Copy BUSCOs	894	91.4
Complete Duplicated BUSCOs	56	5.7
Fragmented BUSCOs	5	0.5
Missing BUSCOs	23	2.4

### Gene prediction and annotation

Before predicting genes, transposable elements (TEs) in the genome were identified using homology-based (RepeatMasker^31^, RepeatScout^32^, RepBase^33^, and RMBlast^34^) and by *de novo* using RepeatModeler^35^. Tandem Repeats Finder^36^ was used to predict consensus sequences and to gain classification information for each repeat. Annotation of repetitive elements resulted in 313,966,700 bp of repetitive DNA, amounting to 43.87% of the genome assembly (Table 3). The majority of the repetitive elements were unclassified (20.41%), followed by Simple repeats (7.38%), SINEs (5.55%), LINEs (4.83%), and DNA elements (3.81%). By using the SSRMMD tool^37^, we identified 1,518,868 simple sequence repeats (SSRs) distributed throughout the genome (Supplementary Table 4). A total of 3,304,085 SNPs has been detected in the *B. attramentaria* genome (Supplementary Table 5), after aligning sequence reads with the BWA tool^38^ and using bcftools^39^ to identify variants. Repetitive elements in the genome were masked before proceeding with the gene prediction. We used EvidenceModeller gene predicting tool for predicting protein-coding genes from the draft genome by combining evidence from *ab initio* gene predictions, transcripts, and protein homologues. We used Augustus^40^ for *ab initio* gene prediction. Additional supports for gene prediction came from two different data sets of transcripts generated by Trinity^41^ from our previous study by Ho *et al*.^22^ and homologous protein sequences of related species to *B. attramentaria* by PASA^42^ and Exonerate^43^. Finally, we used EVidenceModeller^42^ to merge and improve the *ab initio* predictions with the evidence of transcripts and protein sequences with weights of evidences. The predicted genes were annotated using InterProScan with Pfam^44^. A sensitive HMM scanning method on the known Pfam functional domains with an e-value of 0.05 was also used to classify the gene families. Kyoto Encyclopedia of Genes and Genomes (KEGG) annotation was performed using the KEGG Automatic Annotation Server (https://www.genome.jp/kegg/kaas/)^45^ with the bi-directional best hit (BBH) method. Homology-based and *ab initio*-based gene prediction resulted in the identification of 40,596 protein-coding genes (i.e., a total of 29.8% of the genome) with an average gene length of 5,248 bp from the *B. attramentaria* genomes (Table 4). Functional annotation of all predicted protein-coding genes by InterpRoscan resulted in 15,756 (38.8%) genes by Pfam and 17,922 (44.1%) genes by Gene Ontology^46^. A total of 11,074 (27.3%) genes were annotated by KEGG database^46^.

**Table 4:** Statistics of predicted protein-coding genes of *B. attramentaria*.

Feature	Number of features	Total length (bp)	Average length (bp)
Gene	40,596	213,066,832	5,248
CDS	40,596	41,324,867	1,018
Exon	205,270	41,324,867	201
Intron	164,674	172,071,313	1,045

### Phylogenomics

We performed an extensive comparison of orthologous genes among 19 gastropod genomes (*Batillaria attramentaria*, *Conus consors*^28^, *Lanistes nyassanus*^47^, *Marisa cornuarietis*^47^, *Pomacea canaliculata*^47^, *Aplysia californica*, *Elysia chlorotica*^48^, *Plakobranchus ocellatus*^49^, *Biomphalaria glabrata*^50^, *Bulinus truncates*^51^*, Achatina immaculata*^21^, *Lottia gigantea*^52^, *Chrysomallon squamiferum*^53^, *Haliotis rubra*^54^, *Crassostrea gigas*^55^, *Agropecten purpuratus*^56^, and *Octopus bimaculoides*^57^) using OrthoFinder v3.0^58^. With all species present, 3,532 orthogroups were formed, with 36 of those consisting of one-copy genes. With the fasttree tool provided in OrthoFinder, we constructed a tree of rooted species using 573 orthogroups, where at least 81.8% of species had single-copy genes in any orthogroup with *Octopus bimaculoides* as the outgroup. Divergence time was calculated using the species tree generated by using RelTime methods in MEGA-X^59^ with the Jones–Taylor–Thornton model (Fig. 2a). The timetree was computed using two calibration constraints with confidence interval (CI) of *Haliotis rubra*–*Chrysomallon squamiferum* (414–596.9 MYA) and of *Elysia chlorotica*–*Aplysia californica* (58.3–278.9 MYA) that were taken from the TimeTree database^60^ for the calibration of time trees. The divergence time between *B. attramentaria* and *C. consors* was approximately 230 MYA, i.e., during the Early Mesozoic era.

### Comparative genomic analysis

**Fig. 3:**
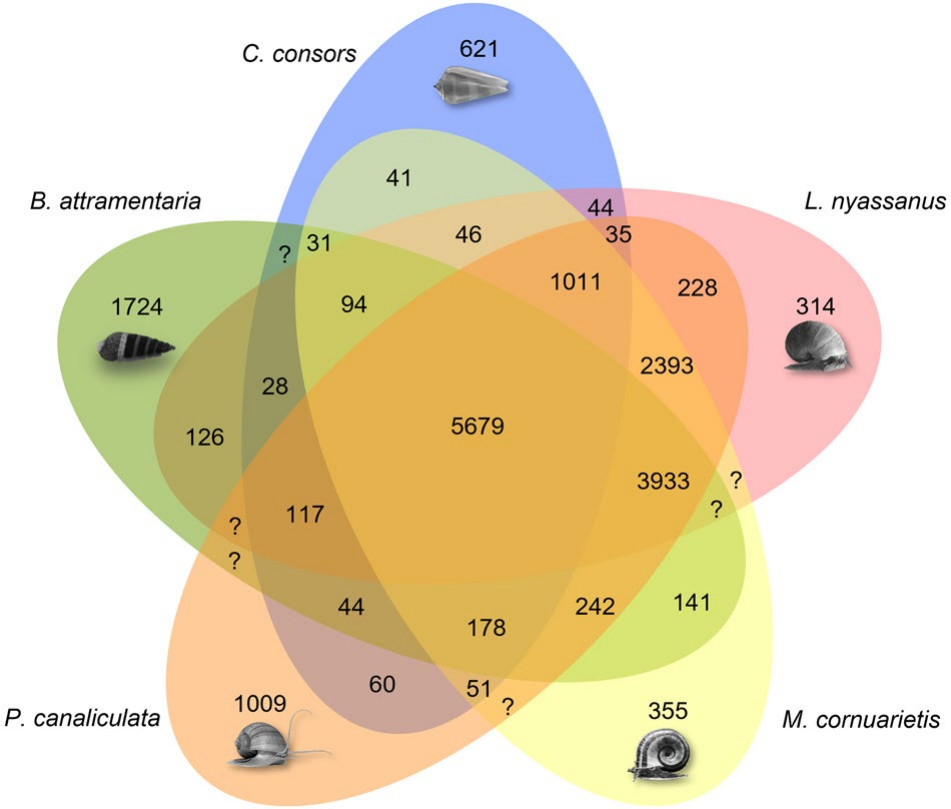
Venn diagram showing the amount of common orthologous gene clusters shared among *B. attramentaria* and its closet relative mollusks including *C. consors*, *L. nyassanus*, *M. cornuarietis*, and *P. canaliculata*.

A comparison of orthologous gene groups shared among related gastropods of *C. consors*, *L. nyassanus*, *M. cornuarietis*, and *P. canaliculata* analysis by OrthoVenn2^61^ showed a core set of 5,679 gene groups and a unique set of 1,724 gene groups was specific to *B. attramentaria* (Fig. 3). Gene ontology (GO) enrichment analysis of the gene groups unique to *B. attramentaria* showed the top five over-representation of GO terms mostly related to protein poly-ADP-ribosylation, GTP binding, and innate immune response (Supplementary Table 6).

## Data Records

All DNA and RNA raw reads have been deposited in the NCBI SRA. All short and long read DNA sequences are available under the NCBI SRA accession number SRP269996^62^, genome assembly with accession number GCA_018292915.1^63^ and the whole genome shotgun sequencing project was deposited in GenBank accession JACVVK000000000^64^ under the BioProject no. PRJNA640962. Supplementary materials which include all supplementary tables, results of comparative genomics and phylogenomic analysed by OrthoFinder, SNPs and SSRs are deposited to Figshare repository^46^: 10.6084/m9.figshare.22309195.v4.

## Technical Validation

### Quality assessment of the DNA and purification

High-quality DNA with bands around and above 10 kb in the agarose gel was selected for sequencing. The quality of the genomic DNA was measured using Bioanalyzer 2100 (Agilent Technologies, CA, USA), and the quantity was measured by a NanoDrop-1000 microspectrophotometer.

### Sequencing read quality validation

FastQC quality control (http://www.bioinformatics.bbsrc.ac.uk/projects/fastqc/) was used to assess the quality of raw high-throughput DNA sequencing datasets. Low-quality sequence PE-reads (<Q20) were filtered out by v.0.32^65^ before assembly^46^.

### Gene prediction and annotation validation

Final gene model prediction of the *B. attramentaria* genome assembly were considered by Evidence Modeler and assessed with the BUSCO (metazoa_odb10). The final predicted gene set consisted of 40,596 genes with (Table 4) with BUSCO value of 81.6%.

## Supplementary information

Supplementary Information

## Data Availability

In this study, software tools used according to the description mentioned in the materials and method section. No custom code was used.
