# Supplementary material for: Genome assembly of the Korean intertidal mud-creeper Batillaria attramentaria
Source: Sci Data. 2023 Jul 28;10:498. doi: 10.1038/s41597-023-02403-9 (PMC10382545; doi:10.1038/s41597-023-02403-9)
Supplement: Supplementary file 1 — Supplementary Information [file 41597_2023_2403_MOESM1_ESM.docx]

**Supplementary Information**

**Genome assembly of the Korean intertidal mud-creeper *Batillaria attramentaria***

### Authors

Ajit Kumar Patra^1^, Phuong-Thao Ho^1,2,3^, Siyeong Jun^1^, Seung Jae Lee^4^, Yuseob Kim^1 †^, and Yong-Jin Won^1^ ^†^

**Affiliations**

1. Department of Life Science, Division of EcoScience, Ewha Womans University, Seoul, South Korea

2. Laboratory of Ecology and Environmental Management, Science and Technology Advanced Institute, Van Lang University, Ho Chi Minh City, Vietnam

3. Department of International Program, US Vietnam Talent International School, Ho Chi Minh city, Viet Nam

4. Bioinformatics Team, DNA Link, Seoul, South Korea

^†^corresponding author(s): Yuseob Kim (yuseob@ewha.ac.kr) and Yong-Jin Won (won@ewha.ac.kr)

**Supplementary Table 1.** List of genes in *B. attramentaria* genome responsible for intertidal adaptation.

| **Category** | **Pfam ID** | **Pfam product** | **Copy of genes** |
| --- | --- | --- | --- |
| Ionic regulation | PF00999 | Sodium/hydrogen exchanger family | 7 |
|  | PF01699 | Sodium/calcium exchanger protein | 12 |
|  | PF02690 | Na+/Pi-cotransporter | 2 |
|  | PF00287 | Sodium / potassium ATPase beta chain | 10 |
|  | PF00690 | Cation transporter/ATPase, N-terminus | 7 |
|  | PF00689 | Cation transporting ATPase, C-terminus | 7 |
|  | PF13246 | Cation transport ATPase (P-type) | 10 |
|  | PF02374 | Anion-transporting ATPase | 1 |
| Osmotic regulation | PF01553 | Acyltransferase | 13 |
|  | PF16076 | Acyltransferase C-terminus | 6 |
|  | PF01757 | Acyltransferase family | 16 |
|  | PF01619 | Proline dehydrogenase | 2 |
|  | PF00208 | Glutamate/Leucine/Phenylalanine/Valine dehydrogenase | 1 |
|  | PF02668 | Taurine catabolism dioxygenase TauD, TfdA family | 8 |
| Aerial Exposure | PF13405 | EF-hand domain | 14 |
|  | PF13499 | EF-hand domain pair | 75 |
|  | PF00209 | Sodium:neurotransmitter symporter family | 51 |
|  | PF01699 | Sodium/calcium exchanger protein | 12 |
|  | PF01545 | Cation efflux family | 17 |
|  | PF02738 | Molybdopterin-binding domain of aldehyde dehydrogenase | 13 |
|  | PF01315 | Aldehyde oxidase and xanthine dehydrogenase, a/b hammerhead domain | 12 |
|  | PF08241 | Methyltransferase domain | 17 |
|  | PF08242 | Methyltransferase domain | 1 |
|  | PF13383 | Methyltransferase domain | 8 |
|  | PF13489 | Methyltransferase domain | 4 |
|  | PF13649 | Methyltransferase domain | 11 |
|  | PF13679 | Methyltransferase domain | 3 |
|  | PF13847 | Methyltransferase domain | 10 |
|  | PF00394 | Multicopper oxidase | 11 |
|  | PF07731 | Multicopper oxidase | 12 |
|  | PF07732 | Multicopper oxidase | 17 |
| Terrestrial respiratory function | PF14830 | Haemocyanin beta-sandwich | 35 |
|  | PF07700 | Haem-NO-binding | 8 |
|  | PF03098 | Animal haem peroxidase | 22 |
|  | PF00080 | Copper/zinc superoxide dismutase (SODC) | 3 |
|  | PF00245 | Alkaline phosphatase | 8 |
|  | PF00782 | Dual specificity phosphatase, catalytic domain | 24 |
|  | PF00459 | Inositol monophosphatase family | 5 |
|  | PF12456 | Inositol phosphatase | 2 |
|  | PF00102 | Protein-tyrosine phosphatase | 68 |
|  | PF16891 | Serine-threonine protein phosphatase N-terminal domain | 3 |
|  | PF02127 | Aminopeptidase I zinc metalloprotease (M18) | 1 |
| Developmental stability | PF02574 | Homocysteine S-methyltransferase | 8 |
|  | PF01596 | O-methyltransferase | 6 |
|  | PF10294 | Lysine methyltransferase | 5 |
|  | PF01571 | Aminomethyltransferase folate-binding domain | 3 |
|  | PF04072 | Leucine carboxyl methyltransferase | 2 |
|  | PF00028 | Cadherin domain | 43 |
|  | PF08266 | Cadherin-like | 10 |
|  | PF12733 | Cadherin-like beta sandwich domain | 2 |
|  | PF05118 | Aspartyl/Asparaginyl beta-hydroxylase | 9 |
|  | PF02373 | JmjC domain, hydroxylase | 7 |
|  | PF00011 | Hsp20/alpha crystallin family | 18 |
|  | PF00012 | Hsp70 protein | 5 |
|  | PF00183 | Hsp90 protein | 2 |
|  | PF00144 | Beta-lactamase | 14 |
|  | PF00753 | Metallo-beta-lactamase superfamily | 8 |
|  | PF16661 | Metallo-beta-lactamase superfamily domain | 4 |
| Aestivation | PF00821 | Phosphoenolpyruvate carboxykinase C-terminal P-loop domain | 2 |
|  | PF17297 | Phosphoenolpyruvate carboxykinase N-terminal domain | 2 |
|  | PF00349 | Hexokinase | 4 |
|  | PF16078 | 2-oxoglutarate dehydrogenase N-terminus | 1 |
|  | PF16870 | 2-oxoglutarate dehydrogenase C-terminal | 3 |
|  | PF02786 | Carbamoyl-phosphate synthase L chain, ATP binding domain | 4 |
|  | PF00988 | Carbamoyl-phosphate synthase small chain, CPSase domain | 1 |
|  | PF00491 | Arginase family | 2 |
| Immune response | PF06990 | Galactose-3-O-sulfotransferase | 15 |
|  | PF01762 | Galactosyltransferase | 33 |
|  | PF02709 | N-terminal domain of galactosyltransferase | 8 |
|  | PF01471 | Putative peptidoglycan binding domain | 4 |
|  | PF01546 | Peptidase family M20/M25/M40 | 8 |
|  | PF13450 | NAD(P)-binding Rossmann-like domain | 8 |
|  | PF00652 | Ricin-type beta-trefoil lectin domain | 18 |
|  | PF01390 | SEA domain | 16 |
|  | PF00095 | WAP-type (Whey Acidic Protein) 'four-disulfide core' | 12 |
|  | PF00386 | C1q domain | 143 |
|  | PF00008 | EGF-like domain | 73 |
|  | PF00629 | MAM domain, meprin/A5/mu | 82 |
|  | PF00229 | TNF (Tumour Necrosis Factor) family | 11 |
|  | PF00020 | TNFR/NGFR cysteine-rich region | 10 |
|  | PF00059 | Lectin C-type domain | 191 |
|  | PF00822 | PMP-22/EMP/MP20/Claudin family | 32 |
|  | PF00811 | Ependymin | 18 |
|  | PF00092 | von Willebrand factor type A domain | 80 |
|  | PF00093 | von Willebrand factor type C domain | 16 |
|  | PF00094 | von Willebrand factor type D domain | 37 |

**Supplementary Table 2.** Sequencing libraries and data yield from Illumina DNA and RNA sequencing of *B. attramentaria*.

|  | **Library Type** | **Insert Size (bp)** | **Read Length (bp)** | **Raw Bases (bp)** | **Raw reads** | **Trimmed Bases (bp)** | **Trimmed Reads** | **SRA accessions** |
| --- | --- | --- | --- | --- | --- | --- | --- | --- |
| DNA | Paired-End (PE) | 350 | 101 | 73,853,334,332 | 731,221,132 | 57,538,068,850 | 569,683,850 | SRX8666490 |
| RNA | Paired-End (PE) | 180 | 101 | 12,859,237,584 | 127,319,184 | 12,289,232,296 | 123,198,958 | SRX2957286 |
|  |  |  |  | 13,244,629,142 | 131,134,942 | 12,699,606,049 | 127,251,498 | SRX2957287 |
|  |  |  |  | 10,875,664,244 | 107,679,844 | 10,391,590,836 | 104,134,796 | SRX2957284 |
|  |  |  |  | 12,285,653,736 | 121,640,136 | 11,748,097,201 | 117,739,554 | SRX2957279 |
|  |  |  |  | 11,364,212,758 | 112,516,958 | 10,888,706,519 | 109,125,782 | SRX2957278 |
|  |  |  |  | 14,226,212,388 | 140,853,588 | 13,629,737,864 | 136,566,862 | SRX2957288 |
|  |  |  |  | 11,265,622,416 | 111,540,816 | 10,775,275,392 | 107,992,744 | SRX2957281 |
|  |  |  |  | 12,215,466,816 | 120,945,216 | 11,679,763,933 | 117,084,540 | SRX2957289 |
|  |  |  |  | 11,698,415,092 | 115,825,892 | 11,213,464,381 | 112,327,814 | SRX2957280 |
|  |  |  |  | 12,769,848,140 | 126,434,140 | 12,193,813,590 | 122,326,650 | SRX2957283 |
|  |  |  |  | 11,609,703,762 | 114,947,562 | 11,104,001,261 | 111,338,558 | SRX2957282 |
|  |  |  |  | 13,213,469,834 | 130,826,434 | 12,664,349,603 | 126,824,892 | SRX2957285 |

**Supplementary Table 3.** Sequencing libraries and data yield from Illumina DNA and RNA sequencing of *B. attramentaria*.

| **Read Type** | **Number of bases** | **Number of reads** | **N50 read length** | **Mean read length** | **SRA accessions** |
| --- | --- | --- | --- | --- | --- |
| Polymerase Reads | 53,338,933,376 | 5,633,914 | 15,250 | 9,467 | SRX8666491 |
| Subreads | 53,287,913,645 | 6,131,297 | 13,923 | 8,691 |  |

**Supplementary Table 4.** Number of SSRs loci of per length of motif of *B. attramentaria* genome.

| **Motif length(bp)** | **SSR number** | **Percentage (%)** |
| --- | --- | --- |
| 1 | 588199 | 38.73 |
| 2 | 661678 | 43.56 |
| 3 | 129346 | 8.52 |
| 4 | 102825 | 6.77 |
| 5 | 31703 | 2.09 |
| 6 | 5117 | 0.34 |
| total | 1518868 | 100 |

**Supplementary Table 5.** Summary of SNPs detected from the *B. attramentaria* genome.

| **Key** | **Value** |
| --- | --- |
| number of samples: | 1 |
| number of records: | 4331495 |
| number of no-ALTs: | 0 |
| number of SNPs: | 3304085 |
| number of MNPs: | 0 |
| number of indels: | 1027410 |
| number of others: | 0 |
| number of multiallelic sites: | 74386 |
| number of multiallelic SNP sites: | 1609 |
| ts | 1855055 |
| tv | 1450639 |
| ts/tv | 1.28 |

**Supplementary Table 6.** GO enrichment of *B. attramentaria* specific unique genes compared with its closet relative mollusks including *C. consors*, *L. nyassanus*, *M. cornuarietis*, and *P. canaliculata* analyzed by OrthoVenn2.

| **Term** | **Count** | **Name** | **Category** | ***P-value*** |
| --- | --- | --- | --- | --- |
| GO:0070212 | 6 | protein poly-ADP-ribosylation | Biological process | 9.02E-06 |
| GO:0005525 | 9 | GTP binding | Molecular function | 4.40E-05 |
| GO:0045087 | 6 | innate immune response | Biological process | 0.000201365 |
| GO:0009103 | 4 | lipopolysaccharide biosynthetic process | Biological process | 0.000212064 |
| GO:0071625 | 4 | vocalization behavior | Biological process | 0.000212064 |
| GO:0048147 | 3 | negative regulation of fibroblast proliferation | Biological process | 0.000251186 |
| GO:0015074 | 4 | DNA integration | Biological process | 0.000432601 |
| GO:0048678 | 3 | response to axon injury | Biological process | 0.000827632 |
| GO:0009617 | 7 | response to bacterium | Biological process | 0.000891502 |
| GO:0010847 | 2 | regulation of chromatin assembly | Biological process | 0.002605186 |
